# A comprehensive analysis of the genomic and proteomic profiles of a megalocytivirus isolated from *Larimichthys crocea*

**DOI:** 10.3389/fmicb.2025.1528930

**Published:** 2025-03-03

**Authors:** Xiaodong Liu, Hongshu Chi, Xixi Yang, Zaiyu Zheng, Chunhua Zhu, Yunkun Wu, Wei-Jen Chang, Hui Gong

**Affiliations:** ^1^Institute of Biotechnology, Fujian Academy of Agricultural Sciences, Fuzhou, China; ^2^Fujian Engineering Technology Research Center for Aquatic Diseases Control and Prevention, Fuzhou, China; ^3^Institute of Animal Husbandry and Veterinary Medicine, Fujian Academy of Agricultural Sciences, Fuzhou, China; ^4^Department of Biology, Hamilton College, Clinton, NY, United States; ^5^State Key Laboratory of Mariculture Breeding, Ningde, China

**Keywords:** genome, megalocytivirus, *Larimichthys crocea*, GO analysis, proteome, electron microscopy, phylogenetic analysis

## Abstract

**Introduction:**

The prevalence of viral diseases has posed significant challenges to the sustainable development of large yellow croaker (*Larimichthys crocea*) aquaculture, with megalocytivirus being one of the primary viral pathogens affecting this species. There have been two proteomic and genomic studies regarding two members of the genus *Megalocytivirus*: the spotted knifejaw iridovirus (SKIV) and the infectious spleen and kidney necrosis virus (ISKNV). However, both studies were conducted more than 10 years ago. To further investigate the pathogenesis of megalocytivirus, we sequenced the genome of the viral strain FD201807 isolated from *L. crocea*, and conducted a proteomics analysis.

**Methods:**

Viral DNA was sequenced using the Illumina HiSeq 2000 platform. Viral proteins from purified virions and supernatants of viral infected cells were subjected to LC-MS/MS analysis, and the expression of four viral proteins was further confirmed by Western blotting. The entire viral genome was subjected to phylogenetic and bioinformatic analyses.

**Results:**

The FD201807 genome comprises 112,214 bp of double-stranded DNA with a G + C content of 53.53%. It contains 130 potential open reading frames, with coding capacities ranging from 41 to 1,293 amino acids. Phylogenetic analysis of the whole-genome sequence indicated that the closest known megalocytivirus related to FD201807 is Pompano iridovirus, with a sequence identity of 98.98%. Label-free proteomics analysis identified 27 viral proteins in the viral-infected cell culture supernatants and 46 viral proteins in the purified virus of FD201807. Among these, 19 viral proteins were detected in both the viral-infected cell culture supernatants and the purified virus samples, while 8 viral proteins were exclusively identified in the viral-infected cell culture supernatants. Notably, there were two proteins derived from the cultured cell line MFF-1 (mandarin fish fry cell line-1), namely cytochrome c and ubiquitin-activating enzyme E1, present in both the purified virus samples and the culture supernatant of infected cells. These cellular proteins may be associated with virus-host protein interactions and/or host cell apoptosis.

**Discussion:**

We present the most comprehensive proteomic analysis to date of the megalocytivirus isolated from *L. crocea*, and help identify highly expressed proteins that may serve as future targets for immunotherapy and biochemical analysis.

## 1 Introduction

The large yellow croaker (*Larimichthys crocea*) is the largest marine fish species under aquaculture in China. In 2023, the annual production of large yellow croaker reached 280,997 tons, accounting for 13.66% of the national marine fish production (Wang et al., 2024). However, in recent years, viral diseases, particularly those caused by megalocytivirus from the family Iridoviridae, have increasingly posed a threat to the sustainable development of marine fish aquaculture, including *Larimichthys crocea*, in China.

The family Iridoviridae is a collection of large icosahedral viruses with double-stranded DNA that are classified into two subfamilies: *Alphairidovirinae* and *Betairidovirinae*. The former comprises three genera (*Ranavirus, Megalocytivirus and Lymphocystivirus*), whose members infect primarily bony fish, amphibians, and reptiles (Chinchar et al., 2017). A survey conducted by Wang and colleagues revealed that megalocytivirus was detected in over 50 cultured and wild marine fish species in the South China Sea (Wang et al., 2007). Iridovirid diseases exhibit a high incidence, with mortality rates reaching up to 75% in young *L. crocea*. Chen et al. confirmed that the epidemic deaths among *L. crocea* fry in the Ningde and Luoyuan districts of Fujian Province from 1999 to 2001 were attributed to iridovirid infections (Chen et al., 2003). Based on its epidemiological, histopathological, and morphological characteristics, this virus was named the large yellow croaker iridovirus (LYCIV). Since then, iridovirid infections in *L. crocea* have been detected across various farming areas (Wang et al., 2006, 2022), underscoring iridovirid diseases as a significant concern for the species.

To date, the genomes of 24 strains within the genus *Megalocytivirus* have been completely sequenced, with sizes ranging from 110 to 131 kbp. As large virus genome databases expand, understanding the expression, function, and regulation of the proteome encoded by these genomes becomes increasingly important. Insights into the protein composition of infectious viral particles, or virions, are essential for functional investigations (Van Vliet et al., 2009). Viral proteomics analysis aids in elucidating viral structure and determining modifications in protein expression during viral attachment and invasion. However, research on the protein functions of iridovirids that infect *L. crocea* has been limited due to a lack of suitable cell lines for viral culture. Ao et al. initially reported the sequencing of the LYCIV genome, providing annotations for a portion of its genes (Ao and Chen, 2006). Recently, Wang et al. published the complete genome sequence of LYCIV-ZS-2020 and established the virus's pathogenicity in large yellow croaker through artificial infection trials (Wang et al., 2022). Nevertheless, the proteome of the large yellow croaker iridovirus has not yet been reported. To date, the only megalocytivirus proteomes reported have been from two other strains: the spotted knifejaw iridovirus (SKIV) (Shuang et al., 2013) and the infectious spleen and kidney necrosis virus (ISKNV) (Dong et al., 2011).

In this study, we present the complete genome of a recently characterized Megalocytivirus strain, FD201807, which was isolated in 2018 from Ningde, Fujian, China. We subjected the purified virus and the cell culture supernatant to protein identification through liquid chromatography–mass spectrometry/mass spectrometry (LC-MS/MS)-based label-free proteomics. This study integrates genomic and proteomic analyses to identify immunogenic structural proteins and membrane proteins that may play a crucial role in viral invasion, in order to help us better understand the infection mechanisms of megalocytivirus and lays the groundwork for developing subunit vaccines against this virus.

## 2 Materials and methods

### 2.1 Virus origin, cultivation and observation

The virus FD201807 was isolated from the kidney of a 3-month-old juvenile *L. crocea* exhibiting illness in 2018. Sequencing of the major capsid protein (MCP) gene has demonstrated that FD201807 belongs to the family Iridoviridae and has been classified as a member of the genus *Megalocytivirus*. FD201807 was maintained and propagated in the mandarin fish fry cell line-1 (MFF-1) (Dong et al., 2008). Specifically, MFF-1 cells, provided by Sun Yat-sen University, were inoculated into 75-cm^2^ cell culture flasks and cultured in Dulbecco's modified Eagle's medium (DMEM), which contained 10% fetal bovine serum (FBS), 100 U/mL penicillin, and 100 μg/mL streptomycin, at 27°C in the presence of 5% CO_2_. After establishing the confluent monolayer, 60 μL of the FD201807 viral suspension, with the viral loads of 2.5 × 10^7^ copies/μL, was added. And the viral culture was maintained until an 80% cytopathic effect was observed in the MFF-1 cells. For electron microscopy, the infected cells were digested with 0.25% trypsin for 1 min, then harvested and re-suspended in a fixative solution (0.1 M cacodylate buffer containing 2.5% glutaraldehyde, pH = 7.4), after a centrifugation at 1,800 rpm for 5 min, the cells were immersed in the fixative solution. Ultrathin sections were prepared and graphed with a transmission electron microscope (JEOL JSM-6380LV, Tokyo, Japan).

### 2.2 Virus purification

The virus-infected cells, stored at −80°C, were subjected to three freeze-thaw cycles. Following this, the cells were centrifuged at 8,000 rpm and 4°C for 40 min, and the supernatant was collected. Precipitation was achieved by centrifugation at 150,000 g and 4°C for 2 h, after which the pellet was resuspended in 0.01 M phosphate-buffered saline (PBS, pH 7.4). Subsequently, 2 mL of 60%, 2 mL of 50%, 2 mL of 40%, 2 mL of 35%, and 1 mL of 30% sucrose solutions were added stepwise from the bottom to the top of ultracentrifuge tubes. The viral suspension was then gently layered on top of the sucrose gradient solution. Further centrifugation was performed at 200,000 g and 4°C for 4 h, followed by the collection of the virus within the 50% sucrose gradient range. This procedure was repeated for two additional rounds of sucrose gradient centrifugation at 150,000 g for 1 h at 4°C. The supernatant was discarded, and the pellet was resuspended in 1 mL of PBS. Finally, the viral suspension underwent 10 min of ultrasonication in an ice bath (power: 200 W, ultrasound: 2 s, interval: 6 s) before being stored at −80°C.

### 2.3 Sodium dodecyl sulfate-polyacrylamide gel electrophoresis (SDS-PAGE)

Purified FD201807 virus proteins (40 μg) were analyzed using SDS-PAGE. A Mini-PROTEAN II cell system (Bio-Rad, Hercules, CA, U.S.A.) with a 5% stacking gel and a 12% separating gel was employed for discontinuous denaturing gel electrophoresis. Following staining with Coomassie Brilliant Blue R-250, the gel was thoroughly destained, scanned, and the image was saved. The SDS-PAGE band sizes were estimated using Quantity One software (Bio-Rad, U.S.A.), based on the sizes of the molecular weight markers.

### 2.4 Genomic DNA extraction

A total of 200 μL of purified virus suspension was utilized for the extraction of viral genomic DNA using the viral genome DNA extraction kit (Tiangen Biotech Co. Ltd., Beijing), in accordance with the manufacturer's protocols. The extracted viral DNA was subsequently eluted with double-distilled water (ddH_2_O) and stored at −20°C.

### 2.5 Sequencing, assembly, and analysis of FD201807 genome

The extracted viral DNA was submitted to Sangon Biotech (Shanghai) Co., Ltd. for library construction and next-generation sequencing using the Illumina Hiseq 2000 (Illumina, San Diego, CA, USA). The initial sequencing data underwent quality assessment via FastQC 0.11.2 (https://www.bioinformatics.babraham.ac.uk/projects/fastqc/). Quality trimming of the Illumina sequencing data was performed using Trimmomatic 0.36 (Bolger et al., 2014), resulting in a refined dataset. For de novo assembly of the Illumina sequencing data, SPAdes 3.5.0 (Bankevich et al., 2012) was utilized. Following the completion of assembly, GapFiller 1.11 (Boetzer and Pirovano, 2012) was employed to fill gaps. Sequence correction was carried out using PrInSeS-G 1.0.0 (Massouras et al., 2010), which rectified errors introduced during assembly and addressed small fragment insertions or deletions. This process yielded the FD201807 virus genome. Based on the genome sequencing data, primers were designed using Oligo 6.44 (https://www.oligo.net/downloads.html) to amplify the terminal and contiguous N sequences of the genome for sequencing and cloning purposes (Table S1). The missing portions were supplemented according to the sequencing results.

Infectious spleen and kidney necrosis virus (ISKNV, GenBank accession no. AF371960) serves as a representative strain of the genus *Megalocytivirus*. Utilizing ISKNV as a reference, we aligned the FD201807 genome to this reference using Molecular Evolutionary Genetics Analysis (MEGA) software version 11 (Tamura et al., 2021). Significant differences were identified, and primers were designed for polymerase chain reaction (PCR) amplification and clone validation, culminating in the final FD201807 genome sequence. The circular genome schematic, which illustrates ORF distribution, GC content, and GC skew, was visualized using the BLAST Ring Image Generator (BRIG) version 0.95 (https://sourceforge.net/projects/brig/).

Gene annotation for the viral genome was performed using the Rapid Annotation using Subsystems Technology (RAST) server. Functional gene annotation was achieved through NCBI BLAST (https://blast.ncbi.nlm.nih.gov/Blast.cgi) and comparisons with several databases, including the Nucleotide sequence database (NT), Non-Redundant protein sequence database (NR), Swiss-Prot protein knowledgebase (SWISSPROT), Translated EMBL Nucleotide Sequence Database (TREMBL), clusters of orthologous groups database (COG), and the Kyoto Encyclopedia of Genes and Genomes (KEGG).

### 2.6 Phylogenetic tree construction and analysis

The genome sequence of FD201807 was aligned with complete gene sequences from various megalocytiviruses using the online software MAFFT (version 7.520, https://mafft.cbrc.jp/alignment/software/) (Katoh et al., 2019). A phylogenetic tree was constructed utilizing the neighbor-joining method in MEGA 11 software (Tamura et al., 2021), with a bootstrap test value of 1,000 and default parameters. The evolutionary relationships between the FD201807 genome and those of other megalocytiviruses were evaluated. Detailed information regarding the megalocytivirus genomes used in this study is presented in Table 1.

**Table 1:** Summary of the genomic information of megalocytiviruses.

**Virus**	**Abbreviation**	**Genome Size (bp)**	**G+C%**	**ORFs**	**GenBank accession no**.	**Reference**
Red seabream iridovirus	RSIV	112415	53	116	AB104413	Kurita et al., 2002
Orange-spotted grouper iridovirus	OSGIV	112636	54	121	AY894343	Lu et al., 2005
Infectious spleen and kidney necrosis virus	ISKNV	111362	55	125	AF371960	He et al., 2001
Turbot reddish body iridovirus	TRBIV	110104	55	115	GQ273492	Shi et al., 2010
Rock bream iridovirus	RBIV	112333	53	119	KC244182	Zhang et al., 2014
Banggai cardinalfish iridovirus	BCIV	111920	55	120	MT926123	Unpublished
Giant seaperch iridovirus	GSIV	112565	53	135	KT804738	Wen and Hong, 2016
Pompano iridovirus	PIV	112052	53	121	MK098187	Unpublished
Three spot gourami iridovirus	TSGIV	111591	56	116	MG570132	Unpublished
South American cichlid iridovirus	SACIV	111347	56	116	MG570131	Unpublished
Angelfish iridovirus	AFIV	111127	55	121	MK689685	Unpublished
Scale drop disease virus	SDDV	131122	37	135	OM037668	Fu et al., 2021
Spotted knifejaw iridovirus	SKIV	112489	53	132	ON075463	Unpublished
Large yellow croaker iridovirus	LYCIV	111760	54	126	AY779031	Ao and Chen, 2006
Large yellow croaker iridovirus isolate Zhoushan	LYCIV-ZS-2020	112043	54	120	MW139932	Wang et al., 2022
Megalocytivirus FD201807	FD201807	112214	54	130	OQ475017	This study

### 2.7 Label-free proteomics analysis of FD201807

Viruses from infected cells were purified using sucrose density gradient centrifugation. Proteins from the supernatants of virus-infected cells, and purified viruses were sent to Novogene Biotech Co., Ltd. (Beijing, China) for label-free proteomics analysis. Total protein was extracted as follows: the sample was treated with an appropriate volume of protein lysis buffer (100 mM ammonium bicarbonate, 8 M urea, 0.2% SDS, pH 8.0) followed by 5 min of ultrasound treatment in an ice-water bath. After centrifugation at 12,000 g and 4°C for 15 min, the supernatant was collected, and 10 mM dithiothreitol (DTT) was added to react at 56°C for 1 h. Subsequently, an adequate amount of iodoacetamide was added, and the mixture was allowed to react at room temperature in the dark for 1 h. The sample was then precipitated with four volumes of pre-cooled acetone at −20°C for 3 h, followed by centrifugation at 12,000 g and 4°C for 15 min to collect the precipitate. The precipitate was washed with 1 mL of pre-cooled acetone and centrifuged at 12,000 g and 4°C for 15 min. Following this, the precipitate was collected, air-dried, and subsequently dissolved in an appropriate volume of protein solubilization buffer (6 M urea, 100 mM triethyl ammonium bicarbonate, pH 8.5). After assessing the protein quality, the sample underwent trypsin digestion and subsequent LC-MS/MS analysis. Mass spectrometry was performed using a data-dependent acquisition mode, with a full scan range from m/z 350 to 1,500 and a resolution of 60,000 (at m/z 200). The resolution for the second-level mass spectrum was set to 15,000 (at m/z 200). The resulting raw data from MS detection were designated as “.raw” files. These raw files were then imported into Proteome Discoverer 2.2 software for retrieval from the UniProt database and for peptide and protein quantification. Additional filtering was conducted using Proteome Discoverer 2.2 to enhance the quality of the analysis results and minimize the false-positive rate. Peptide spectrum matches (PSMs) with a credibility exceeding 99% were identified. Proteins that contained at least one unique peptide were classified as credible proteins. Only credible peptides and proteins were retained, and false discovery rate (FDR) validation was performed to exclude peptides and proteins with an FDR >1%.

### 2.8 Bioinformatics analysis

Gene Ontology (GO) annotation was performed by conducting sequence alignment (BLAST) of the identified protein sequences against six widely recognized databases: protein family databases (Pfam), Protein fingerprint database (PRINTS), Protein domain database (ProDom), Simple Modular Architecture Research Tool (SMART), Protein site database (ProSite), and Protein Analysis Through Evolutionary Relationships (PANTHER). This was followed by analysis using the InterProScan software (http://www.ebi.ac.uk/interpro/search/sequence/).

### 2.9 Western blotting

Freeze-thaw infected cells were separated using 12% SDS-PAGE gels and subsequently transferred onto a polyvinylidene fluoride (PVDF) membrane. Polyclonal antibodies raised in mice against the purified recombinant proteins (VP007L, VP058L, and VP121R) were employed as primary antibodies at a dilution of 1:1,000. Additionally, a monoclonal antibody targeting VP026R, generously provided by Sun Yat-sen University, was also utilized as a primary antibody at the same dilution of 1:1,000. The PVDF membrane was then subjected to immunodetection using a secondary antibody conjugated with horseradish peroxidase (HRP) at a dilution of 1:10,000 (Proteintech, Wuhan, China) and was visualized through enhanced chemiluminescence.

### 2.10 Virus abbreviations

The names of viruses in this article are abbreviated as follows: ISKNV for Infectious spleen and kidney necrosis virus; PIV for Pompano iridovirus; RSIV for Red seabream iridovirus; LYCIV for Large yellow croaker iridovirus; LYCIV-ZS-2020 for Large yellow croaker iridovirus isolate Zhoushan; OSGIV for Orange-spotted grouper iridovirus; RBIV for Rock bream iridovirus; TRBIV for Turbot reddish body iridovirus; GSIV for Giant seaperch iridovirus; SACIV for South American cichlid iridovirus; SKIV for Spotted knifejaw iridovirus; TSGIV for Three spot gourami iridovirus; BCIV for Banggai cardinalfish iridovirus; AFIV for Angelfish iridovirus; and LMIV for Lateolabrax maculatus iridovirus.

## 3 Results

### 3.1 Transmission electron microscopy of viral particles

**Figure 1:**
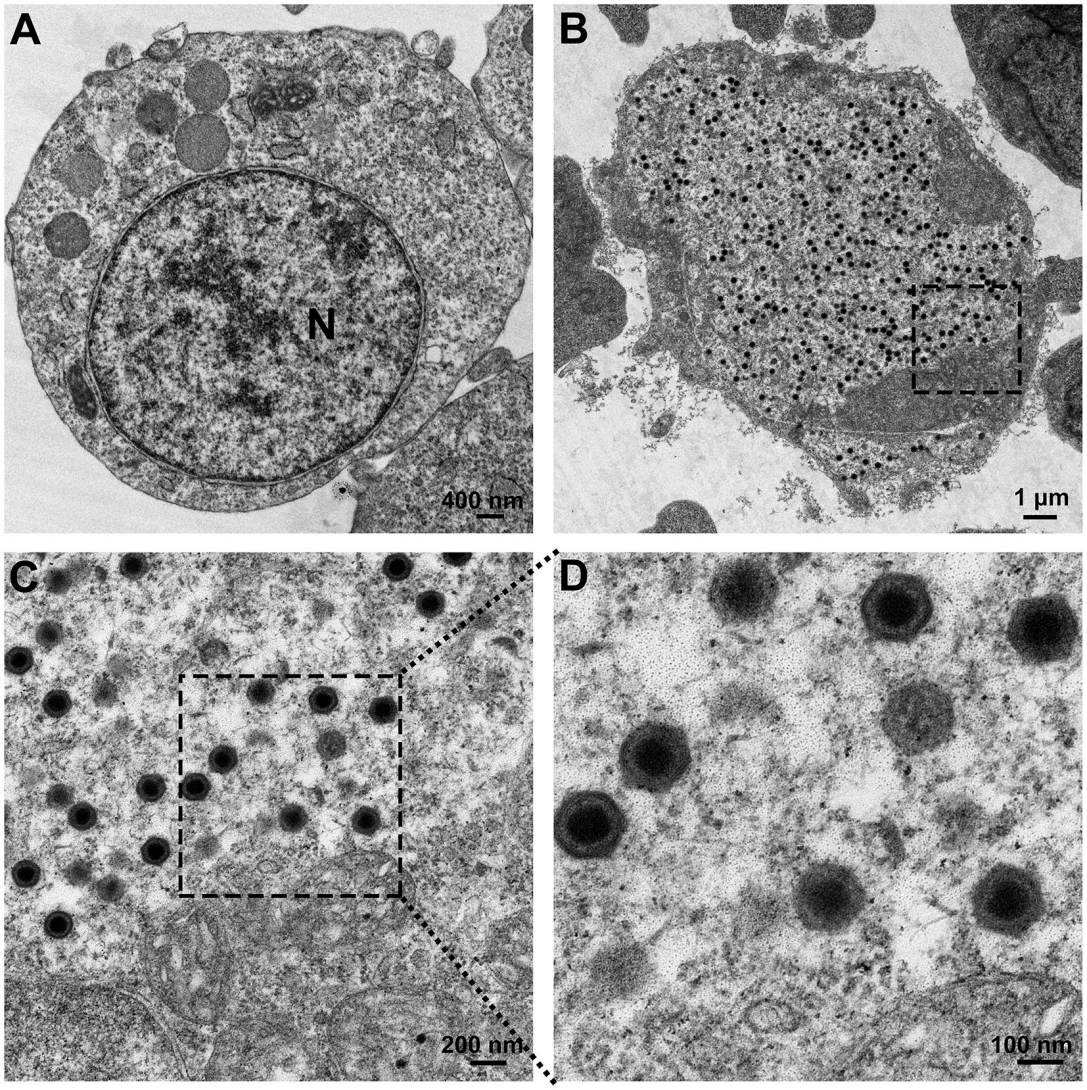
Electron microscopic observation of the virus. **(A)** Transmission electron micrograph of normal MFF-1 cells (scale bar = 400 nm). **(B)** Ultrastructure of MFF-1 cells after infection with FD201807 (scale bar = 1 μm). **(C)** Higher magnification of the virions observed in **(B)** (scale bar = 200 nm). **(D)** Higher magnification of the virions observed in **(C)** (scale bar = 100 nm).

Transmission electron microscopy was conducted on FD201807-infected cells, which exhibited a pathological rate of 80%. Compared to normal MFF-1 cells (Figure 1A), abundant viral particles were observed, scattered randomly within the cytoplasm of the infected cells (Figure 1B). FD201807-infected cells also appeared larger in size than the normal MFF-1 cell, as indicated by the differences in scale bar between Figures 1A, B, which aligns with a key symptom of Megalocytivirus infection. Upon magnification, viral particles and some empty capsids with a roughly hexagonal shape and bilayer envelope were visible, exhibiting a uniform size with a diameter of approximately 150 nm (Figures 1C, D).

### 3.2 Determination of the FD201807 genome sequence

Illumina HiSeq sequencing yielded 20,709,962 reads with a total base count of 3,106,494,300 bp. After Tirmmomatic clipping, 19,852,088 reads, or 2,753,330,175 bp were retained, with a Q20 base rate of 97.76%. A genome of 111,110 bp was assembled by processing these reads through SPAdes for assembly (> 24,000 × coverage), GapFiller for gap completion, and PrInSeS-G for sequence correction. The ends of the viral sequences were amplified using PCR, and the sequences were determined by Sanger sequencing. The final, complete genome of FD201807 is 112,214 bp in length with a G + C content of 53.53%, slightly lower than that of ISKNV (54.78%). NCBI BLAST genome comparison demonstrated that the FD201807 viral genome shared over 90% identity with 20 other genomes from the genus *Megalocytivirus*. Analysis of the genome termini indicated that the FD201807 genome DNA is both circularly permuted and terminally redundant, characteristics commonly shared among members of the Iridoviridae family (Chinchar et al., 2017).

### 3.3 ORFs and their predicted protein products

**Figure 2:**
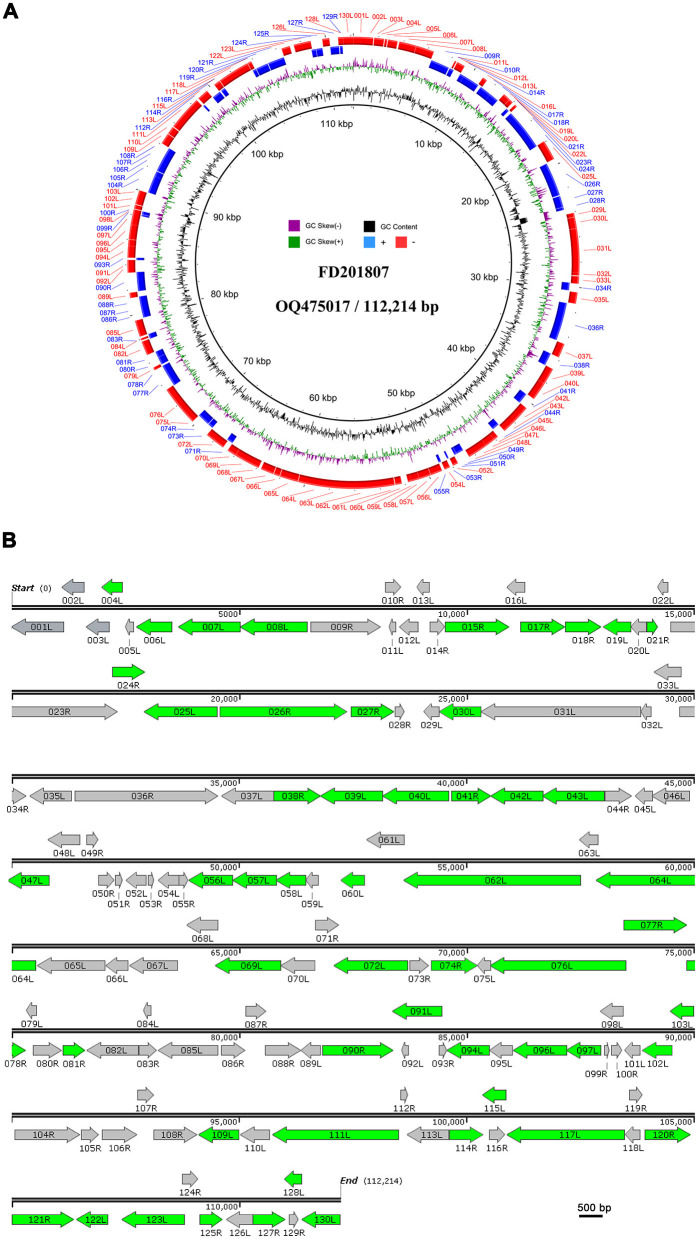
Circular representation and organization of FD201807. **(A)** Circos plot of FD201807. The FD201807 genome encoded 130 predicted proteins. From the outer circle to the inner circle, the figure shows predicted ORFs (red: negative chain; blue: positive chain), GC skew curve (green: positive GC skew; violet: negative GC skew), and GC content (black). **(B)** Arrows represent ORFs with respect to their size, location, and orientation. The green arrows represent proteins identified by LC-MS/MS.

We identified 130 putative ORFs encoding proteins with lengths ranging from 41 to 1,293 amino acids on both the sense (R) and antisense (L) DNA strands. Among these ORFs, 52 (40%) were transcribed in the forward orientation, whereas 78 (60%) were oriented in the reverse orientation (Figure 2). Each ORF's hypothetical amino acid sequence was BLAST-searched against the GenBank nr database. All ORFs matched homologous sequences within GenBank's Megalocytivirus entries, with 97 aligning fully with registered Megalocytivirus protein sequences. A substantial majority (65.4%, or 85) of FD201807 ORFs lacked predicted functions. Of the 130 ORFs, 44 encoded proteins with homology to functionally annotated proteins, and all 26 iridovirid core genes were identified. These core genes are believed to play essential roles in viral transcription, replication, and assembly (Eaton et al., 2007).

The putative gene products of FD201807 included at least 10 enzymes involved in transcription, nucleotide metabolism, and DNA replication. These included DNA-dependent RNA polymerase subunit H (ORF002L), ribonucleotide-diphosphate reductase subunit beta (ORF027R), the two largest subunits of DNA-dependent RNA polymerase (ORF031L and ORF036R), transcription elongation factor S-II (TFIIS, ORF032L), mRNA capping enzyme (ORF065L), deoxyribonucleoside kinase (ORF034R), D5 family NTPase (ORF111L), DNA polymerase (ORF023R), sucrose non-fermenting 2 (SNF2) family helicase (ORF064L), proliferating cell nuclear antigen (PCNA, ORF114R), and one RNase III enzyme (ORF088R). These FD201807 genes exhibited 94–97% amino acid sequence identity to their homologs in ISKNV (Dong et al., 2011). Detailed annotation information for each ORF is provided in Table S2.

Some inferred gene products of FD201807 contained highly conserved structural domains and enzymatic active site motifs involved in protein processing and modification. For instance, ORF015R featured a serine/threonine protein kinase domain, ORF026R contained three calcium-binding epidermal growth factor (EGF)-like domains, and ORF117L included a tyrosine-specific protein phosphatase active site.

Notably, FD201807 displayed a unique feature on the positive strand at positions 8,313–8,319, consisting of seven adenine residues, unlike other Megalocytivirus strains, which typically have eight consecutive adenine residues. This deletion was confirmed by a separate PCR followed by Sanger sequencing, indicating a one-adenosine deletion likely resulting in a truncated ORF011L and a unique ORF010R (Figure S1).

### 3.4 Phylogenetic analysis

**Figure 3:**
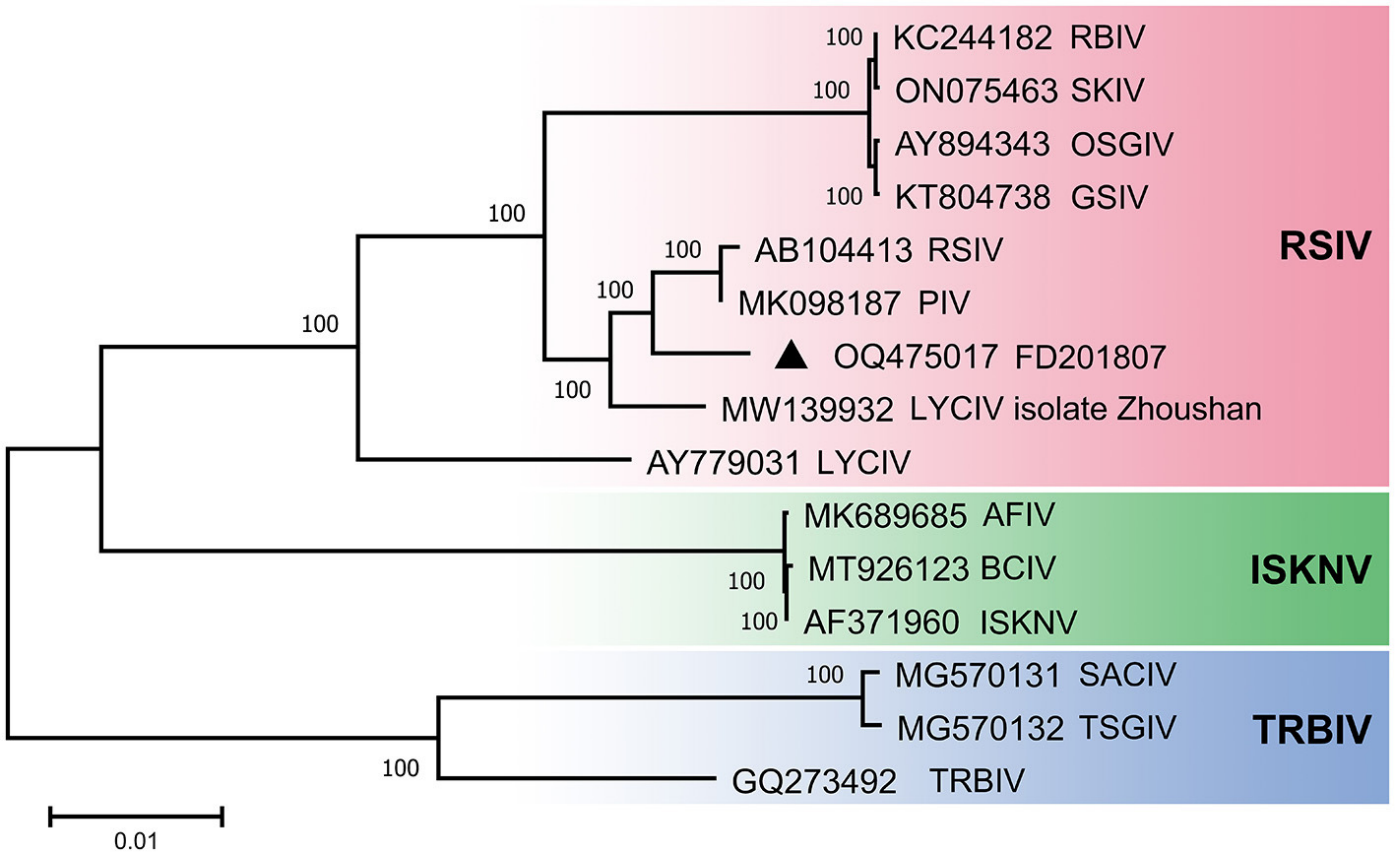
Phylogenetic analyses of megalocytivirus FD201807. Neighbor-Joining tree derived from comparing full-length nucleotide sequences of FD201807 to 14 other megalocytiviruses. Branch supports are indicated by values derived from 1000 bootstrapping steps. The scale bar represents the genetic distance.

A phylogenetic tree was constructed using the complete genome sequences of megalocytiviruses within the family Iridoviridae that are available in GenBank (Figure 3). The results of the phylogenetic analysis indicated that FD201807 is classified within the RSIV genotype and is closely related to PIV and RSIV, exhibiting sequence identities of 98.98 and 98.92%, respectively. Other members of the RSIV genotype include RBIV, SKIV, OSGIV, GSIV, RSIV, LYCIV, and LYCIV-ZS-2020. Notably, two LYCIV strains are genetically distinct: one was isolated from Zhoushan in 2020 (LYCIV-ZS-2020), and the other from the Ningde area of Fujian Province in 2006 (LYCIV) (Ao and Chen, 2006). Our findings suggest that LYCIV-ZS-2020, FD201807, RSIV, and PIV form a sister clade to a group comprising four closely related megalocytiviruses: RBIV, SKIV, OSGIV, and GSIV (Figure 3). The earliest isolated strain of LYCIV (Ao and Chen, 2006) appears to be the earliest diverging member of the RSIV genotype. Additionally, AFIV, BCIV, and ISKNV constitute a monophyletic ISKNV genotype, while SACIV, TSGIV, and TRBIV are categorized under the TRBIV genotype.

### 3.5 Identification of FD201807 proteins using LC-MS/MS and western blotting

**Figure 4:**
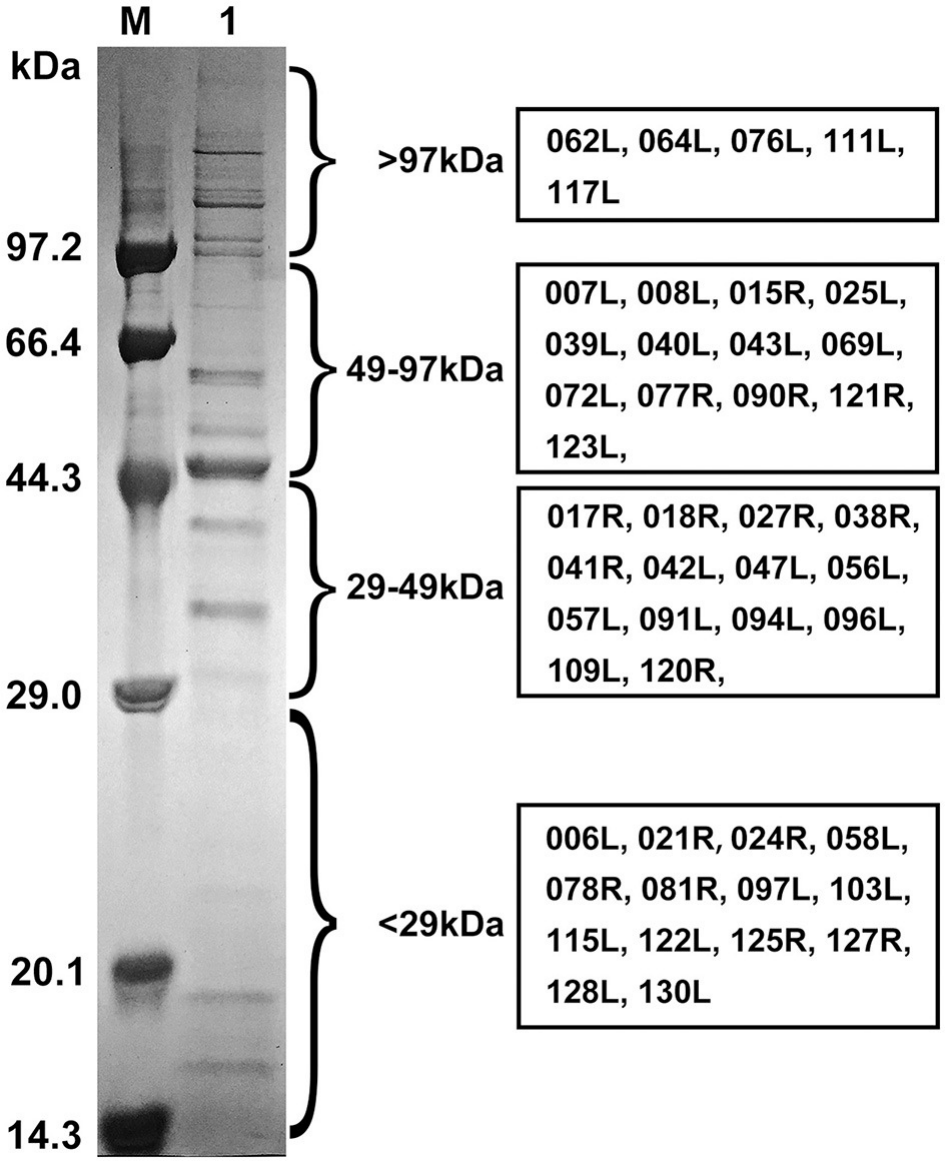
SDS-PAGE profile and regions of FD201807 virion proteins based on the LC-MS/MS identification. M, Protein molecular weight marker (Low) (TaKaRa, Dalian, China); 1, purified FD201807 virion proteins. FD201807 proteins were separated by 12% one-dimensional SDS-PAGE and stained with Coomassie brilliant blue R-250. The viral protein lane was divided into four slices, which, based on the comparison with a molecular marker, ranged from >97 kDa, 49–97 kDa, and 29–49 kDa to <29 kDa. Proteins were in-gel-digested with trypsin, extracted, and subjected to LC-MS/MS. The boxes on the right provide the ORF numbers of the identified proteins.

The purified viral particles were subjected to SDS-PAGE analysis, which revealed approximately 22 distinct protein bands stained with Coomassie brilliant blue. Based on the protein sizes obtained by LC-MS/MS analysis, we indicate the regions where various viral proteins are located in Figure 4. Notably, primary viral proteins were concentrated in bands with molecular weights above 29 kDa, while proteins below 29 kDa showed lower abundance (Figure 4). Trypsin digestion followed by LC-MS/MS analysis identified 595 peptide segments from both the purified virus and cell culture supernatant, corresponding to 141 proteins (Table S3). Most peptide segments ranged from 8 to 18 amino acids in length, with a majority of proteins exhibiting molecular weights between 10 and 60 kDa (Figure S2). Amino acid sequences were searched against the Uniprot and GenBank databases using BLAST, leading to the identification of 54 viral proteins after excluding duplicates. Of these, 47 relatively abundant proteins contained multiple peptide fragments, while smaller or low-abundance proteins, such as VP004L, VP019L, and VP021R, contained only one identifiable peptide (Table 2).

**Table 2:** Virion-associated viral proteins of FD201807 identified by LC-MS/MS.

**No**.	**Protein name (ORF)**	**Coverage (%)**	**Protein ID**	**Matched peptides number**	**Predicted structure and/or function**	**Homology to megalocytiviral ORF**		
						**RSIV**	**ISKNV**	**TRBIV**
1	004L	6	Q4KSF3	1	No hit	390R	003L	004L
2	**006L**	10	A0A140G0I4	2	NLI interacting factor-like phosphatase	385R	005L	005L
3	**007L**	67	E5FQ63, A0A023GU66, E7DRK4, V5QQM2, V5QRG8, I6NLP5, Q1X6P6, A0A191ZDL4	72	major capsid protein	380L	006L	006L
4	**008L**	42	A0A140GBB8, Q8QUV3	27	TM, myristylated membrane protein	374R	007L	007L
5	**015R**	6	A0A140GBB9	3	serine/threonine protein kinase	349L	013R	013R
6	017R	12	A0A140GB23, A0A140G0J0	4	No hit	342L	014R	014R
7	018R	5	M1T4L7	2	No hit	333L	015R	015R
8	019L	9	A0A140G0J2	1	No hit	324R	016L	016L
9	021R	15	A0A140G0J5	1	TM	-	-	018R
10	024R	27	A0A140GB32	14	No hit	-	-	-
11	025L	11	Q5DLW1, Q8B4N1	8	putative phosphatase	292R	022L	023L
12	026R	11	Q5DLW0	6	TM, laminin-type epidermal growth factor-like protein	291L	023R	024R
13	**027R**	7	A0A140G0K2	5	TM, ribonucleotide-diphosphate reductase subunit beta	268L	024R	025R
14	**030L**	9	A0A140GBC8	2	XPG/RAD2 family DNA repair protein	256R	027L	027L
15	038R	20	A0A140GB38	5	No hit	197L	036R	035R
16	039L	38	Q0IKK1	16	No hit	186R	037L	036L
17	040L	23	Q5YF49	11	No hit	180R	038L	037L
18	041R	8	M1S0R7	2	No hit	179L	039R	038R
19	042L	7	A0A140G0M0	2	No hit	171R	040L	039L
20	043L	25	A0A140GB45	7	No hit	162R	041L	040L
21	047L	7	M1T4N8	1	No hit	145R	045L	044L
22	056L	65	M1SQL6	15	2-cysteine adaptor domain-containing protein	111R	054L	051L
23	**057L**	35	A0A140GBD4	16	RGD, 2-cysteine adaptor domain-containing protein	106R	055L	052L
24	**058L**	23	A0A140GB56, Q8QUQ4	5	No hit	101R	056L	053L
25	060L	25	M1SWT0	3	No hit	097R	-	055L
26	062L	18	A0A140G0P0, M1S0T3, A0A140GBD6	42	putative DNA-binding protein	077R	062L	057L
27	**064L**	19	M1T4P8	13	RGD, SNF2 family helicase	063R	063L	058L
28	069L	12	A0A140GB60	5	No hit	033R	068L	063L
29	072L	13	M1S0U3	6	No hit	018R	071L	065L
30	074R	10	M1TCW9	3	No hit	016L	074R	067R
31	**076L**	11	Q4KS82, A0A140G0Q2	14	No hit	639R	076L	069L
32	077R	12	M1S0U7	5	ankyrin repeat containing protein	641L	077R	070R
33	078R	35	Q5YF12	9	No hit	635L	078R	071R
34	081R	72	A0A140GB74	10	No hit	628L	081R	074R
35	090R	8	M1T4R8, Q8QUM2	10	No hit	589L	088R	082R
36	**091L**	9	A0A140GBF4	3	RGD, TM, myristylated membrane protein	575R	90.5L	083R
37	094L	26	A0A140GB82	6	No hit	569R	093L	085L
38	096L	21	A0A140GB84, A0A140G0R5	13	No hit	554R	095L	087L
39	**097L**	22	M1TCY2	13	No hit	550R	096L	088L
40	102L	40	Q5YEZ5	5	No hit	539R	100L	091L
41	103L	81	A0A140GB91, A0A140G0S1	22	No hit	535R	101L	092L
42	109L	3	A0A140GB97	1	No hit	506R	106L	097L
43	**111L**	10	Q4KS51, Q8QUK1	10	D5 family NTPase	493R	109L	099L
44	**114R**	8	Q4KS48	1	proliferating cell nuclear antigen(PCNA)	487L	112R	102R
45	115L	63	A0A140GBA1	16	No hit	-	-	-
46	**117L**	2	M1SQQ5	1	tyrosine kinase	463R	114L	105L
47	**120R**	18	A0A140GBA5, A0A140G0V9	10	RGD, immediate-early protein ICP-46	458L	115R	106R
48	121R	10	M1T4T8	5	No hit	450L	117R	107R
49	122L	38	A0A140G0T3	15	No hit	436R	118L	108L
50	123L	8	K4HMB3	4	ankyrin repeat containing protein	424R	119L	109L
51	125R	8	A0A140G0T6	2	No hit	420L	121R	111R
52	**127R**	11	A0A1L6KVN9	5	ATPase	412L	123R	113R
53	128L	59	A0A140GBB4	11	RGD, TM	407R	-	-
54	130L	41	M1SQR2, G1BEX9	7	RGD, ankyrin repeat containing protein	401R	125L	115L

Core gene associated viral protein are shown in bold.

**Figure 5:**
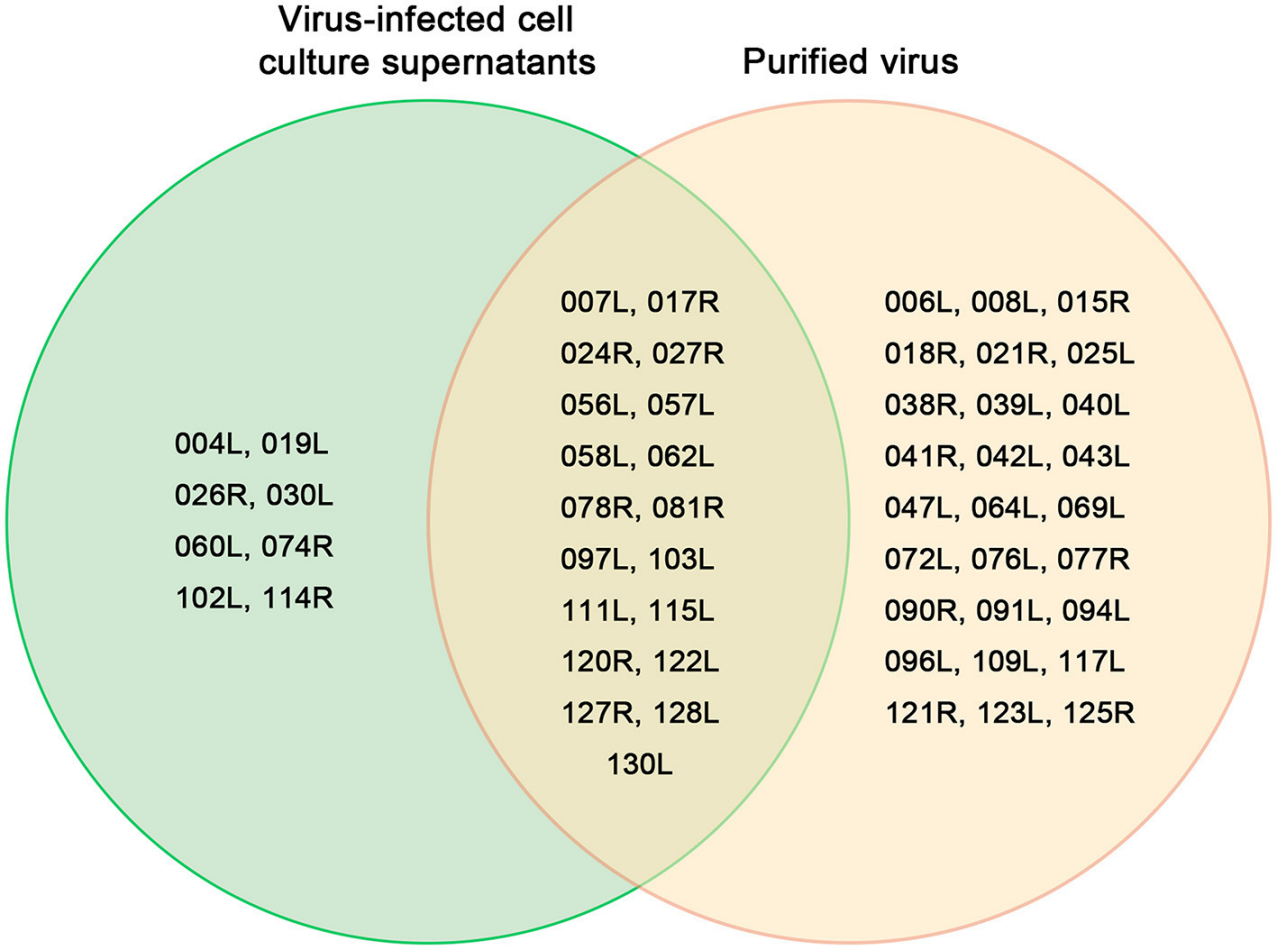
Venn diagram of 54 proteins from the purified virus and the cell culture supernatant identified by LC-MS/MS.

Of the 54 viral proteins, 27 were found exclusively in the purified virus, while 8 proteins (VP004L, VP019L, VP026R, VP030L, VP060L, VP074R, VP102L, and VP114R) were present only in the cell culture supernatant (Figure 5). Additionally, two host-origin proteins, cytochrome c and ubiquitin-activating enzyme E1 were detected in both the MFF-1 cell culture supernatant and the purified virus.

Among the 54 viral proteins, 23 had functional and/or domain annotations, 6 proteins contained transmembrane domains, while another 6, including a SNF2 family helicase (VP064L), had an Arg-Gly-Asp (RGD) motif, which is linked to DNA repair (Table 2). Moreover, FD201807 ORFs 008L, 058L, and 122L are likely to encode envelope proteins, as their putative protein sequences share 97, 99, and 97% identity, respectively, with ISKNV envelope proteins 007L, 056L, and 118L (Dong et al., 2011). Notably, only three proteins (VP007L, VP078R, and VP081R) exhibited acetylation modifications.

**Figure 6:**
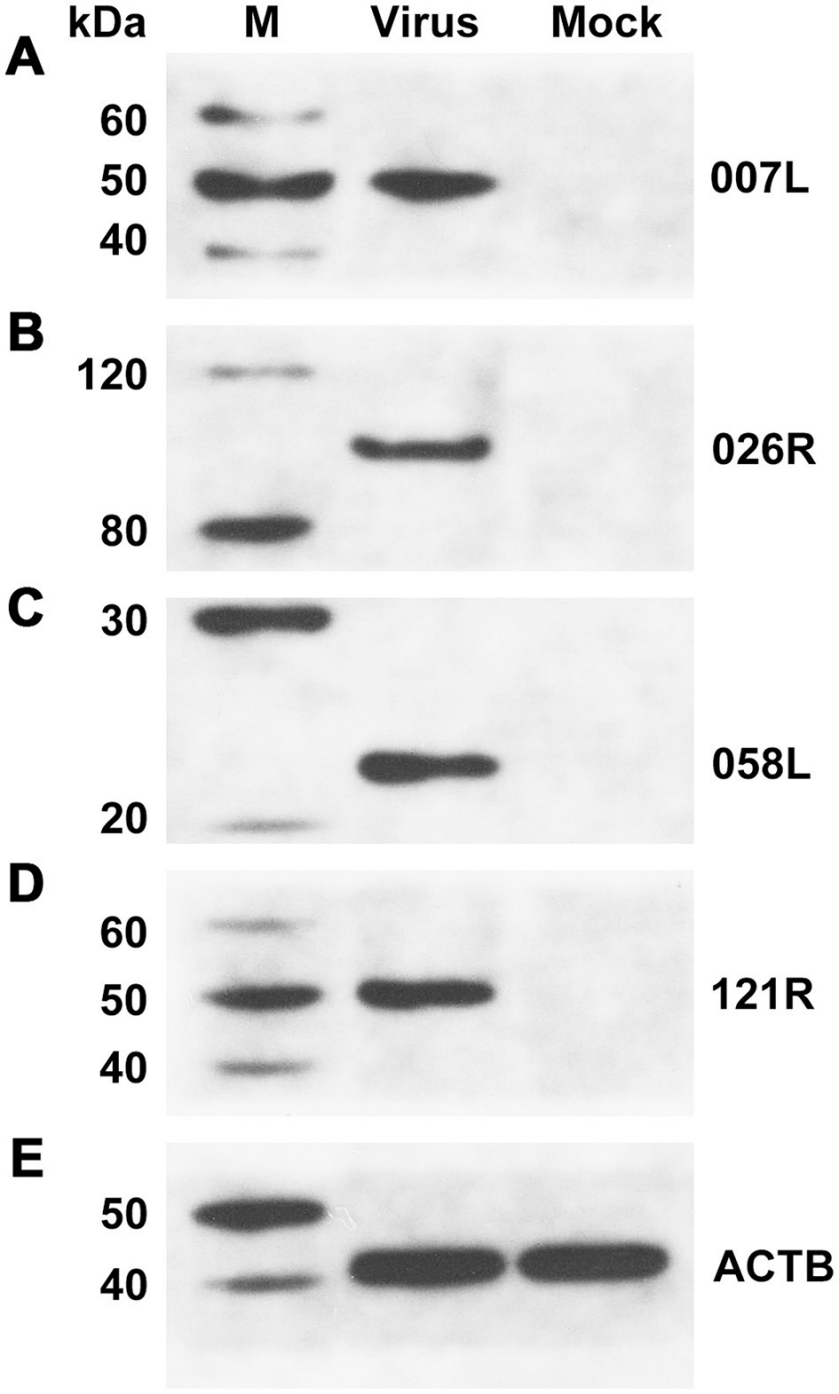
Western blotting confirmed the presence of viral proteins in unpurified viral solution. **(A)** VP007L, **(B)** VP026R, **(C)** VP058L, **(D)** VP121R, and **(E)** ACTB. β-actin served as the internal reference.

To confirm the presence of the identified proteins, we utilized mouse antibodies as the primary antibody in Western blotting for VP007L (49.6 kDa), VP026R (100.8 kDa), VP058L (23.4 kDa), and VP121R (49.6 kDa). Samples containing the virus displayed distinct bands corresponding to the expected protein molecular weights (Figure 6).

### 3.6 GO annotation of identified proteome of FD201807

**Figure 7:**
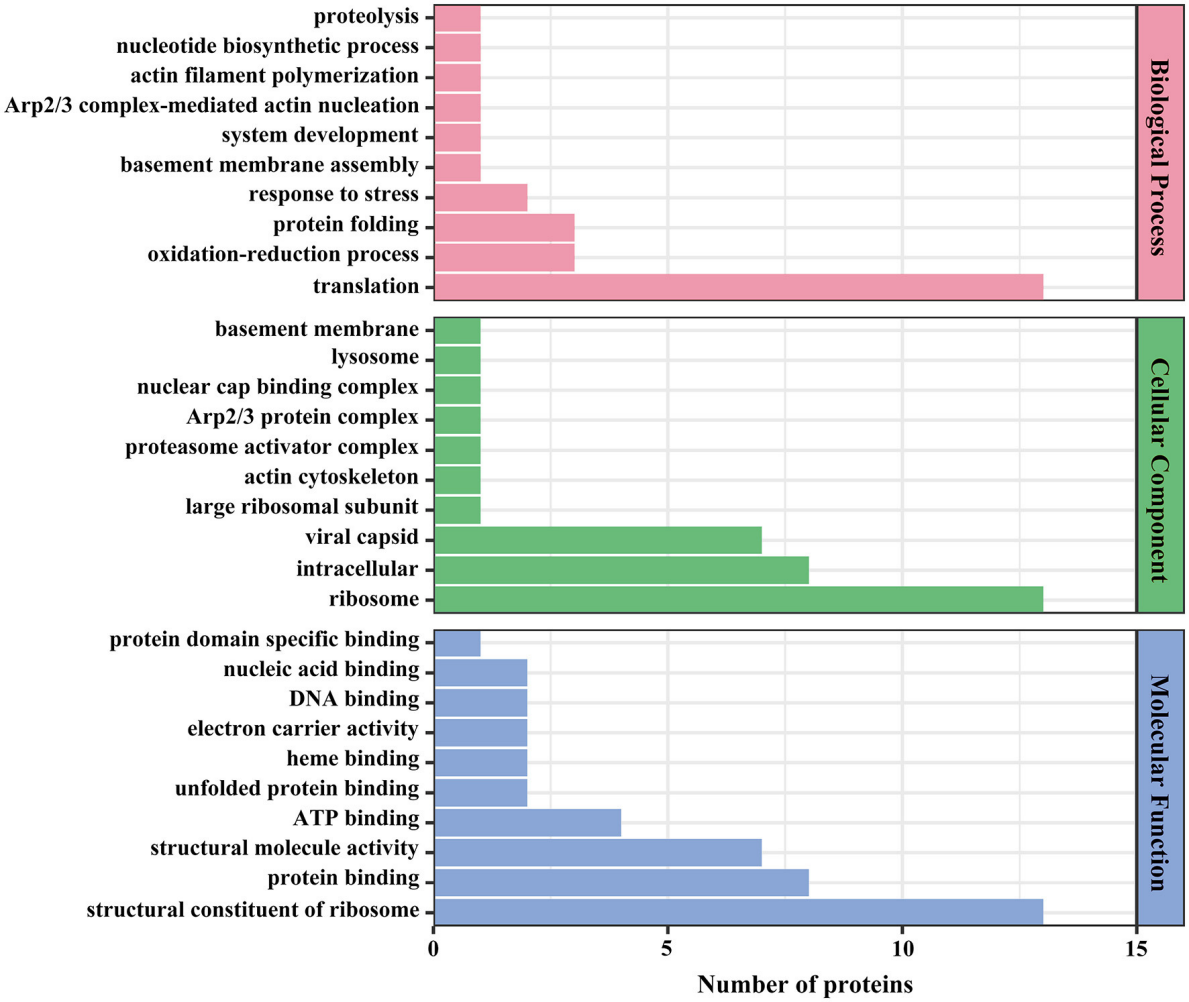
GO annotation of viral proteins. The x-axis denotes the number of proteins, and the y-axis denotes the GO classification.

The 54 identified viral proteins underwent Gene Ontology (GO) classification to help gain insights into functional enrichment across biological processes, cellular components, and molecular functions. The classification of biological processes indicated that viral proteins primarily participate in protein folding, oxidation–reduction, and translation processes. In terms of cellular component, the classification revealed that viral proteins predominantly originated from viral capsids, intracellular locales, and ribosomes. The molecular function classification highlighted roles in ATP binding, structural molecule activity, suggesting their involvement as structural components of ribosomes (Figure 7).

## 4 Discussion

Outbreaks of viral disease caused by megalocytivirus have frequently afflicted young *L. crocea* in the Fujian and Zhejiang regions of China, particularly during the summer months in recent years. With a mortality rates reaching up to 75%, megalocytivirus infections have resulted in substantial economic losses for the *L. crocea* aquaculture industry. This study involved the whole-genome sequencing of megalocytivirus strain FD201807. The purified virus and its cell culture supernatant were analyzed using label-free proteomics methods, and selected viral proteins were further validated through Western blotting.

In this study, a phylogenetic tree was constructed using the complete genome sequence. Phylogenetic tree show that FD201807 is more closely related to PIV and RSIV. In fact, the two LYCIV sequences are distantly related, suggesting the existence of an early-diverging LYCIV genotype (Figure 3).

In previous studies, Dong et al. identified 38 virion-associated proteins from the distantly related ISKNV (Dong et al., 2011), while Shuang et al. identified 49 viral proteins from SKIV (RSIV-type megalocytivirus) (Shuang et al., 2013). In our study, we identified 54 viral proteins from both the virions and the supernatants of virus-infected cells. Notably, two host-origin proteins, ubiquitin-activating enzyme E1 and cytochrome c were detected in both the cell culture supernatant and the purified virus, suggesting that they likely bind to the viral particles and are involved in host-virus interactions. Ubiquitin-activating enzyme E1 is responsible for activating ubiquitin, the first step in ubiquitination. Viruses are known to hijack the host ubiquitin system by encoding ubiquitin ligases, redirecting host ligases, or regulating deubiquitinases, leading to stoichiological changes such as proteasomal degradation, protein relocalization, and regulation of protein or nucleic acid interactions: so-called non-degradative ubiquitination (Herrmann et al., 2020). There is growing evidence that ubiquitin-proteasome system (UPS) is essential for viral infection by affecting virus entry, proliferation, transcription, assembly, release, and immune escape (Wang et al., 2016; Huang et al., 2018; Pang et al., 2021). Cytochrome c (CYC) is a kind of mitochondrial apoptogenic factors (Hu and Yao, 2016). Cytochrome c plays a crucial role in apoptosis: in Anagrapha falcifera multiple nuclear polyhedrosis virus (AfMNPV)-induced apoptosis in Spodoptera litura cells. Inhibiting cytochrome c expression could significantly reduce AfMNPV-induced apoptosis in Sl-1 cells, resulting in decrease of caspase-3 and caspase-9 activity (Liu et al., 2007); the mitochondrial inner membrane protein cytochrome c1 was reported to interact with the nonstructural protein 4 (nsp4) of porcine reproductive and respiratory syndrome virus (PRRSV), which was confirmed by the RNA interference experiments to be pivotal in PRRSV-induced apoptosis (Zhang et al., 2017). Our findings raised potential links between these two pathways and the megalocytivirus, suggesting that cellular apoptosis is worthy of concern in *L. crocea* diseases.

Eight viral proteins (VP004L, VP019L, VP026R, VP030L, VP060L, VP074R, VP102L, and VP114R) were identified exclusively in the supernatant of cell culture and may be secreted or non-structural proteins. FD201807 ORF026R encodes a laminin-type epidermal growth factor–like protein, which is a common ORF among all members of the genus *Megalocytivirus*. Unlike RSIV (AAQ07956), PIV (AZQ20773), LYCIV (ABI32391), RBIV (AAN86692), OSGIV (AAX82335) and LYCIV-ZS-2020 (QQA04019), FD201807 ORF026R contains a 141-nucleotide deletion between positions 21,187 and 21,188, resulting in a truncated protein VP026R (Figure S3). VP23R of ISKNV is homologous to VP026R and, although not a viral envelope protein, is expressed on the plasma membrane of ISKNV-infected cells. ISKNV VP23R interacts with extracellular nidogen-1 to form the virus-mock basement membrane. The heptapeptide sequence “NIDDNPV” within ISKNV VP23R, crucial for its interaction with the basement membrane protein nidogen-1 (Xu et al., 2010), is also present in FD201807 VP026R, guiding future studies on the pathogenic mechanisms of megalocytiviruses. FD201807 VP030L belongs to the XPG/RAD2 family DNA repair protein, and VP114R is a type of proliferating cell nuclear antigen (PCNA), which is a key factor in DNA replication and cell cycle regulation in yeast, plant, and animal cells (Strzalka and Ziemienowicz, 2011). The other five proteins, however, have not been functionally characterized in megalocytiviruses, presenting potential directions for future research.

Proteomics analysis of FD201807 identified 17 of the 26 core gene-associated viral proteins from the family Iridoviridae. Further analysis of the FD201807 genome revealed the other nine undetected core gene-associated viral proteins that included DNA polymerase, DNA-dependent RNA polymerase, deoxyribonucleoside kinase, and Erv1/Alr family proteins (essential for respiration and liver regeneration). These non-structural proteins are involved in DNA and RNA syntheses and regulations. They may have differential expression patterns or could be expressed at levels too low to be detected in this study.

This study enriched the genome and proteome data of large yellow croaker iridovirus, and provided a reference for the study of the infection mechanism of large yellow croaker iridovirus and the development of vaccines.

## Data Availability

The complete genome sequence of megalocytivirus FD201807 was deposited in NCBI under BioProject ID PRJNA931111 and the accession number OQ475017. The mass spectrometry proteomics data have been deposited to the ProteomeXchange Consortium via the PRIDE partner repository with the dataset identifier PXD039383.
