# Supplementary material for: A comprehensive analysis of the genomic and proteomic profiles of a megalocytivirus isolated from Larimichthys crocea
Source: Front Microbiol. 2025 Mar 3;16:1528930. doi: 10.3389/fmicb.2025.1528930 (PMC11911517; doi:10.3389/fmicb.2025.1528930)
Supplement: Supplementary file 1 [file Table_1.docx]

Table S1. The primers for gaps in Hiseq 2000 sequencing of FD201807

| Description | Sequence(5’-3’) |
| --- | --- |
| 43F | TGGGAATTGTCATTGGGTCT |
| 505R | CGCTCTGGCTTCTGGAAT |
| 1183F | AGCATGGAATTTACAATGAATGAAG |
| 1926R | TGATGATAGCGAAAGTGAGTACAAC |
| 7491F | TGTGGCTGGATGTGGCAAACT |
| 8537R | CAACCGCCCGGATGGATT |
| 29521F | AGACTCAGCCGAGCAATACAA |
| 30255R | TATGATGAAGCTTGCCACGC |
| 33583F | GCTTCCGATGCCCGTACT |
| 34091R | ACACGACCCCTGCAAGATG |
| 38155F | CGCAGTACACGTCTTGCC |
| 38568R | CTTTATGCTGGTKCCGAG |
| 41435F | CAAAACAGATGCTGCAAGG |
| 41943R | TGCTACGGAGCTGTACGTG |
| 80843F | CAGGGAGGGCTTAACAGA |
| 81264R | GCACAAGACTGCCAGAGTT |
| 84033F | GTCATTACAGTTTGTTGCGGACAC |
| 84456R | TGGCACCAAGCCGAGCAT |
| 70853F | TCCTCATAGCGTCTACCTTG |
| 71358R | GTTGCCACTTATCGTCCAG |
| 108429F | CTTGAGTTCGGGACCTGC |
| 108968R | ACATGGGCTGACATAGATACATT |
| FD_417F | GATCCTGAGACTGGCGAATG |
| FD_417R | ACCACAGCGGGTGAAACG |
