# Supplementary material for: A comprehensive analysis of the genomic and proteomic profiles of a megalocytivirus isolated from Larimichthys crocea
Source: Front Microbiol. 2025 Mar 3;16:1528930. doi: 10.3389/fmicb.2025.1528930 (PMC11911517; doi:10.3389/fmicb.2025.1528930)
Supplement: Supplementary file 2 [file Table_2.docx]

Table S2. Predicted ORFs of megalocytivirus FD201807

| ORF^a^ | Nucleotide positions | Length  (aa) | Best match(es)^b^ | | | | Predicted motif and/or function |
| --- | --- | --- | --- | --- | --- | --- | --- |
|  |  |  | protein(s) | GenBank  accession no. | BLASTP  score | % aa  identity ^c^ |  |
| 001L | 1-1137 | 378 | LYCIV | ABI32390 | 747 | 100 | transmembrane amino acid transporter protein |
| 002L | 1107-1574 | 155 | PIV | AZQ20750 | 320 | 100 | DNA dependent RNA polymerase subunit H |
| 003L | 1646-2131 | 161 | LYCIV | QQA03998 | 330 | 100 | caspace recruitment domain-containing protein |
| 004L | 1995-2447 | 150 | PIV | AZQ20752 | 307 | 100 |  |
| 005L | 2502-2678 | 58 | PIV | AZQ20753 | 122 | 100 |  |
| 006L | **2752-3516** | **254** | **PIV** | **AZQ20754** | **530** | **100** | **NLI interacting factor-like phosphatase** |
| 007L | **3662-5023** | **453** | **LMIV** | **QBL98278** | **941** | **100** | **major capsid protein** |
| 008L | **5040-6497** | **485** | **PIV** | **AZQ20756** | **990** | **100** | **Myristylated membrane protein** |
| 009R | 6568-8118 | 516 | PIV | AZQ20757 | 1073 | 100 |  |
| 010R | 8214-8561 | 115 | ISKNV | QQZ00464 | 157 | 92.5 |  |
| 011L | 8311-8439 | 42 | PIV | AZQ20758 | 96.7 | 100 |  |
| 012L | 8533-8925 | 130 | RBIV | AAT71826 | 270 | 100 |  |
| 013L | 8922-9182 | 86 | ISKNV | NP_612233 | 176 | 100 |  |
| 014R | 9200-9532 | 110 | PIV | AZQ20761 | 227 | 100 | RING-finger-containing E3 ubiquitin ligase |
| 015R | **9539-10936** | **465** | **PIV** | **AZQ20762** | **966** | **100** | **serine/threonine protein kinase** |
| 016L | 10899-11276 | 125 | PIV | AZQ20763 | 255 | 100 |  |
| 017R | 11191-12165 | 324 | PIV | AZQ20764 | 665 | 99.7 |  |
| 018R | 12171-12962 | 263 | PIV | AZQ20765 | 541 | 100 |  |
| 019L | 13020-13607 | 195 | PIV | AZQ20766 | 408 | 100 |  |
| 020L | 13622-13954 | 110 | PIV | AZQ20767 | 235 | 100 |  |
| 021R | 13960-14217 | 85 | PIV | AZQ20768 | 173 | 100 |  |
| 022L | 14214-14423 | 69 | PIV | AZQ20769 | 117 | 100 |  |
| 023R | **14489-17332** | **947** | **RSIV** | **O70736** | **1979** | **100** | **DNA polymerase** |
| 024R | 17212-17925 | 237 | PIV | AZQ20771 | 486 | 100 |  |
| 025L | 17909-19507 | 532 | PIV | AZQ20772 | 1065 | 98.7 | putative phosphatase |
| 026R | 19580-22375 | 931 | TRBIV | ADE34369 | 1509 | 93.3 | laminin-type epidermal growth factor |
| 027R | **22465-23403** | **312** | **RSIV** | **Q9QTF2** | **652** | **100** | **ribonucleotide-diphosphate reductase subunit beta** |
| 028R | 23427-23642 | 71 | LYCIV | QQA04021 | 298 | 100 |  |

Continued on following page

TABLE S2—Continued

| ORF^a^ | Nucleotide positions | Length  (aa) | Best match(es)^b^ | | | | Predicted motif and/or function |
| --- | --- | --- | --- | --- | --- | --- | --- |
|  |  |  | protein(s) | GenBank  accession no. | BLASTP  score | % aa  identity ^c^ |  |
| 029L | 24069-24392 | 107 | PIV | AZQ20776 | 224 | 100 |  |
| 030L | **24414-25310** | **298** | **PIV** | **AZQ20777** | **622** | **100** | **XPG/RAD2 family DNA repair protein** |
| 031L | **25327-28833** | **1168** | **PIV** | **AZQ20778** | **2433** | **100** | **DNA dependent RNA polymerase alpha subunit** |
| 032L | **28840-29061** | **73** | **PIV** | **AZQ20779** | **152** | **100** | **transcription factor S-II** |
| 033L | 29129-29713 | 194 | PIV | AZQ20780 | 405 | 100 |  |
| 034R | **29689-30321** | **210** | **PIV** | **AZQ20781** | **442** | **100** | **deoxyribonucleoside kinase** |
| 035L | 30407-31309 | 300 | PIV | AZQ20782 | 638 | 100 |  |
| 036R | **31392-34550** | **1052** | **PIV** | **AZQ20783** | **2202** | **99.9** | **DNA dependent RNA polymerase beta subunit** |
| 037L | 34623-35762 | 379 | PIV | AZQ20784 | 785 | 100 |  |
| 038R | 35756-36811 | 351 | PIV | AZQ20785 | 724 | 100 |  |
| 039L | 36808-38157 | 449 | PIV | AZQ20786 | 936 | 100 |  |
| 040L | 38166-39605 | 479 | PIV | AZQ20787 | 997 | 100 |  |
| 041R | 39671-40549 | 292 | PIV | AZQ20788 | 603 | 100 |  |
| 042L | 40542-41681 | 379 | LYCIV | QQA04035 | 769 | 98.2 |  |
| 043L | 41683-43032 | 449 | PIV | AZQ20790 | 927 | 100 |  |
| 044R | 43047-43646 | 199 | PIV | AZQ20791 | 406 | 100 |  |
| 045L | **43730-44092** | **120** | **OSGIV** | **AAX82354** | **248** | **100** | **Erv1 / Alr family protein** |
| 046L | 44099-44899 | 266 | LYCIV | QQA04039 | 548 | 100 |  |
| 047L | 44904-45818 | 304 | PIV | AZQ20794 | 628 | 100 |  |
| 048L | 45812-46495 | 227 | PIV | AZQ20795 | 472 | 100 | cytosine DNA methyltransferase |
| 049R | 46655-46918 | 87 | PIV | AZQ20796 | 177 | 100 |  |
| 050R | 46915-47268 | 117 | PIV | AZQ20797 | 242 | 100 | vascular endothelial growth factor |
| 051R | 47284-47454 | 56 | PIV | AZQ20798 | 116 | 100 |  |
| 052L | 47524-47952 | 142 | PIV | AZQ20799 | 296 | 98.6 |  |
| 053R | 48007-48141 | 44 | PIV | AZQ20800 | 93.6 | 100 |  |
| 054L | 48230-48682 | 150 | PIV | AZQ20801 | 311 | 100 |  |
| 055R | 48684-48899 | 71 | PIV | AZQ20802 | 141 | 100 |  |
| 056L | 48912-49856 | 314 | PIV | AZQ20803 | 646 | 100 | 2-cysteine adaptor domain-containing protein |

Continued on following page

TABLE S2—Continued

| ORF^a^ | Nucleotide positions | Length  (aa) | Best match(es)^b^ | | | | Predicted motif and/or function |
| --- | --- | --- | --- | --- | --- | --- | --- |
|  |  |  | protein(s) | GenBank  accession no. | BLASTP  score | % aa  identity ^c^ |  |
| 057L | **49879-50820** | **313** | **PIV** | **AZQ20804** | **634** | **100** | **2-cysteine adaptor domain-containing protein** |
| 058L | **50831-51478** | **215** | **OSGIV** | **AAX82366** | **432** | **100** |  |
| 059L | 51485-51745 | 86 | PIV | AZQ20806 | 178 | 100 |  |
| 060L | 52251-52763 | 170 | OSGIV | AAX82368 | 348 | 100 |  |
| 061L | **52827-53633** | **268** | **OSGIV** | **AAX82369** | **563** | **100** | **replication factor** |
| 062L | 53630-57511 | 1293 | RBIV | AGG37939 | 2627 | 98.8 | putative DNA-binding protein |
| 063L | 57498-57899 | 133 | RSIV | UUU46929 | 273 | 100 |  |
| 064L | **57877-60525** | **882** | **RBIV** | **AGG37940** | **1835** | **99.6** | **SNF2 family helicase** |
| 065L | 60565-62037 | 490 | PIV | AZQ20812 | 1015 | 100 | mRNA capping enzyme |
| 066L | 62079-62546 | 155 | PIV | AZQ20813 | 315 | 100 | RING-finger-containing E3 ubiquitin ligase |
| 067L | 62596-63639 | 347 | PIV | AZQ20814 | 717 | 100 | RING-finger-containing E3 ubiquitin ligase |
| 068L | 63859-64527 | 222 | PIV | AZQ20815 | 459 | 100 |  |
| 069L | 64478-65911 | 477 | PIV | AZQ20816 | 974 | 100 |  |
| 070L | 65923-66654 | 243 | PIV | AZQ20817 | 496 | 100 |  |
| 071R | 66676-67191 | 171 | PIV | AZQ20818 | 363 | 100 |  |
| 072L | 67094-68704 | 536 | PIV | AZQ20819 | 1097 | 98.7 |  |
| 073R | 68745-69176 | 143 | TRBIV | ADE34411 | 288 | 95.8 |  |
| 074R | 69225-70247 | 340 | TRBIV | ADE34412 | 701 | 97.9 |  |
| 075L | 70256-70525 | 89 | PIV | AZQ20822 | 171 | 100 |  |
| 076L | **70527-73499** | **990** | **LYCIV** | **QQA04069** | **2062** | **99.4** |  |
| 077R | 73461-74852 | 463 | LYCIV | QQA04070 | 918 | 97.6 | ankyrin repeat containing protein |
| 078R | 74849-75313 | 154 | PIV | AZQ20825 | 319 | 100 |  |
| 079L | 75315-75539 | 74 | RBIV | AAT71891 | 151 | 97.3 |  |
| 080R | 75468-76100 | 210 | RBIV | AGG37957 | 441 | 100 |  |
| 081R | 76113-76610 | 165 | OSGIV | AAX82389 | 339 | 99.4 |  |
| 082L | 76659-77783 | 374 | PIV | AZQ20829 | 771 | 99.7 |  |
| 083R | 77786-78184 | 132 | OSGIV | AAX82391 | 278 | 100 |  |
| 084L | 77902-78054 | 50 | ISKNV | NP_612305 | 70.5 | 88 |  |

Continued on following page

TABLE S2—Continued

| ORF^a^ | Nucleotide positions | Length  (aa) | Best match(es)^b^ | | | | Predicted motif and/or function |
| --- | --- | --- | --- | --- | --- | --- | --- |
|  |  |  | protein(s) | GenBank  accession no. | BLASTP  score | % aa  identity ^c^ |  |
| 085L | 78217-79521 | 434 | PIV | AZQ20831 | 899 | 99.8 |  |
| 086R | 79608-80141 | 177 | PIV | AZQ20832 | 367 | 100 |  |
| 087R | **80138-80605** | **155** | **PIV** | **AZQ20833** | **321** | **99.4** |  |
| 088R | **80571-81368** | **265** | **PIV** | **AZQ20834** | **558** | **100** | **RNase III** |
| 089L | 81365-81778 | 137 | PIV | AZQ20835 | 279 | 100 | SAP domain-containing protein |
| 090R | 81827-83401 | 524 | PIV | AZQ20836 | 1058 | 98.1 |  |
| 091L | **83382-84452** | **356** | **RSIV** | **UNA01383** | **716** | **97.5** | **myristylated membrane protein** |
| 092L | 83581-83715 | 44 | OSGIV | AAX82397 | 89.7 | 93.2 |  |
| 093R | 84391-84576 | 61 | OSGIV | AAX82398 | 129 | 100 |  |
| 094L | 84573-85499 | 308 | OSGIV | AAX82399 | 617 | 99.7 |  |
| 095L | 85509-86009 | 166 | PIV | AZQ20839 | 342 | 100 |  |
| 096L | 86034-87197 | 387 | PIV | AZQ20950 | 798 | 100 |  |
| 097L | **87205-87957** | **250** | **RBIV** | **AGG37971** | **520** | **100** |  |
| 098L | 87948-88439 | 163 | PIV | AZQ20842 | 336 | 100 |  |
| 099R | 88033-88158 | 41 | ISKNV | NP_612319 | 77.4 | 92.7 |  |
| 100R | 88183-88434 | 83 | GSIV | AMM72727 | 163 | 98.8 |  |
| 101L | 88489-88812 | 107 | PIV | AZQ20843 | 220 | 100 | RING-finger-containing E3 ubiquitin ligase |
| 102L | 88868-89515 | 215 | PIV | AZQ20844 | 447 | 100 |  |
| 103L | 89484-89999 | 171 | PIV | AZQ20845 | 357 | 100 |  |
| 104R | 90068-91513 | 481 | PIV | AZQ20846 | 1008 | 100 | ankyrin repeat containing protein |
| 105R | 91520-91933 | 137 | PIV | AZQ20847 | 285 | 100 | suppressor of cytokine signaling protein |
| 106R | 91995-92771 | 258 | PIV | AZQ20848 | 536 | 100 |  |
| 107R | 92773-93144 | 123 | PIV | AZQ20849 | 253 | 100 |  |
| 108R | 93126-94091 | 321 | PIV | AZQ20850 | 660 | 99.7 | HIT family protein |
| 109L | 94128-95000 | 290 | LYCIV | QQA04096 | 609 | 100 |  |
| 110L | 95047-95667 | 206 | PIV | AZQ20852 | 431 | 100 |  |
| 111L | **95748-98510** | **920** | **PIV** | **AZQ20853** | **1938** | **100** | **D5 family NTPase** |
| 112R | 98563-98718 | 51 | PIV | AZQ20854 | 97.8 | 100 |  |
| 113L | 98715-99611 | 298 | PIV | AZQ20855 | 607 | 97.7 | tumor necrosis factor receptor-associated factor |

Continued on following page

TABLE S2—Continued

| ORF^a^ | Nucleotide positions | Length  (aa) | Best match(es)^b^ | | | | Predicted motif and/or function |
| --- | --- | --- | --- | --- | --- | --- | --- |
|  |  |  | protein(s) | GenBank  accession no. | BLASTP  score | % aa  identity ^c^ |  |
| 114R | **99631-100374** | **247** | **PIV** | **AZQ20856** | **509** | **100** | **proliferating cell nuclear antigen** |
| 115L | 100364-100867 | 167 | PIV | AZQ20857 | 344 | 100 |  |
| 116R | 100509-100862 | 117 | RSIV | BAK14294 | 226 | 100 |  |
| 117L | **100906-103488** | **860** | **PIV** | **AZQ20858** | **1799** | **100** | **tyrosine kinase** |
| 118L | 103513-103815 | 100 | PIV | AZQ20859 | 194 | 100 |  |
| 119R | 103595-103882 | 95 | SACIV | AVR29759 | 162 | 82.3 |  |
| 120R | **103924-104934** | **336** | **PIV** | **AZQ20860** | **706** | **100** | **immediate-early protein ICP-46** |
| 121R | 104995-106371 | 458 | PIV | AZQ20861 | 920 | 99.1 |  |
| 122L | 106425-107099 | 224 | PIV | AZQ20862 | 462 | 97.8 |  |
| 123L | 107432-108796 | 454 | PIV | AZQ20863 | 943 | 100 | ankyrin repeat containing protein |
| 124R | 108739-109101 | 120 | PIV | AZQ20864 | 249 | 100 | RING-finger-containing E3 ubiquitin ligase |
| 125R | 109131-109637 | 168 | PIV | AZQ20865 | 350 | 100 |  |
| 126L | 109734-110294 | 186 | PIV | AZQ20866 | 387 | 100 |  |
| 127R | **110304-111023** | **239** | **GSIV** | **AAL68653** | **502** | **100** | **ATPase** |
| 128L | 110995-111375 | 126 | GSIV | AMM72753 | 258 | 100 |  |
| 129R | 111101-111313 | 70 | OSGIV | AAX82428 | 125 | 100 |  |
| 130L | 111384-112208 | 274 | PIV | AZQ20869 | 569 | 100 | ankyrin repeat containing protein |

**Bold** show 26 core iridovirid genes

^a^ open reading frame number.

^b^ most closely related gene by BLASTP analysis.

^c^ amino acid percent identity for most closely related protein
