# Supplementary material for: A comprehensive analysis of the genomic and proteomic profiles of a megalocytivirus isolated from Larimichthys crocea
Source: Front Microbiol. 2025 Mar 3;16:1528930. doi: 10.3389/fmicb.2025.1528930 (PMC11911517; doi:10.3389/fmicb.2025.1528930)
Supplement: Supplementary file 3 [file Table_3.docx]

Table S3. Overview of the protein identification

| Name | Total spectra | Matched spectrum | Peptide | Identified protein |
| --- | --- | --- | --- | --- |
| virus | 34,586 | 549 | 346 | 63 |
| supernatant | 39,875 | 306 | 249 | 78 |
