# Supplementary figures and images for: A comprehensive analysis of the genomic and proteomic profiles of a megalocytivirus isolated from Larimichthys crocea
Source: Front Microbiol. 2025 Mar 3;16:1528930. doi: 10.3389/fmicb.2025.1528930 (PMC11911517; doi:10.3389/fmicb.2025.1528930)

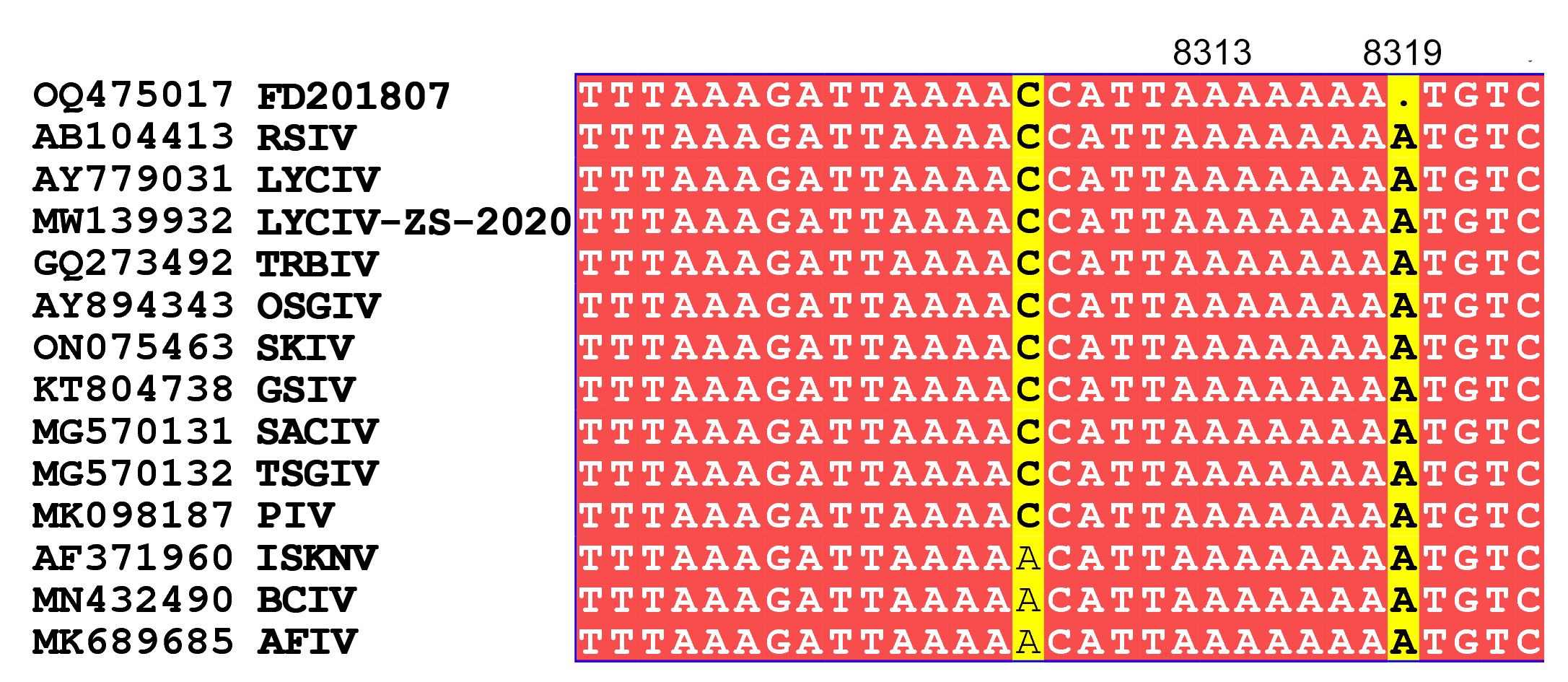

Supplement: Supplementary file 4 [file Image_1.tif]

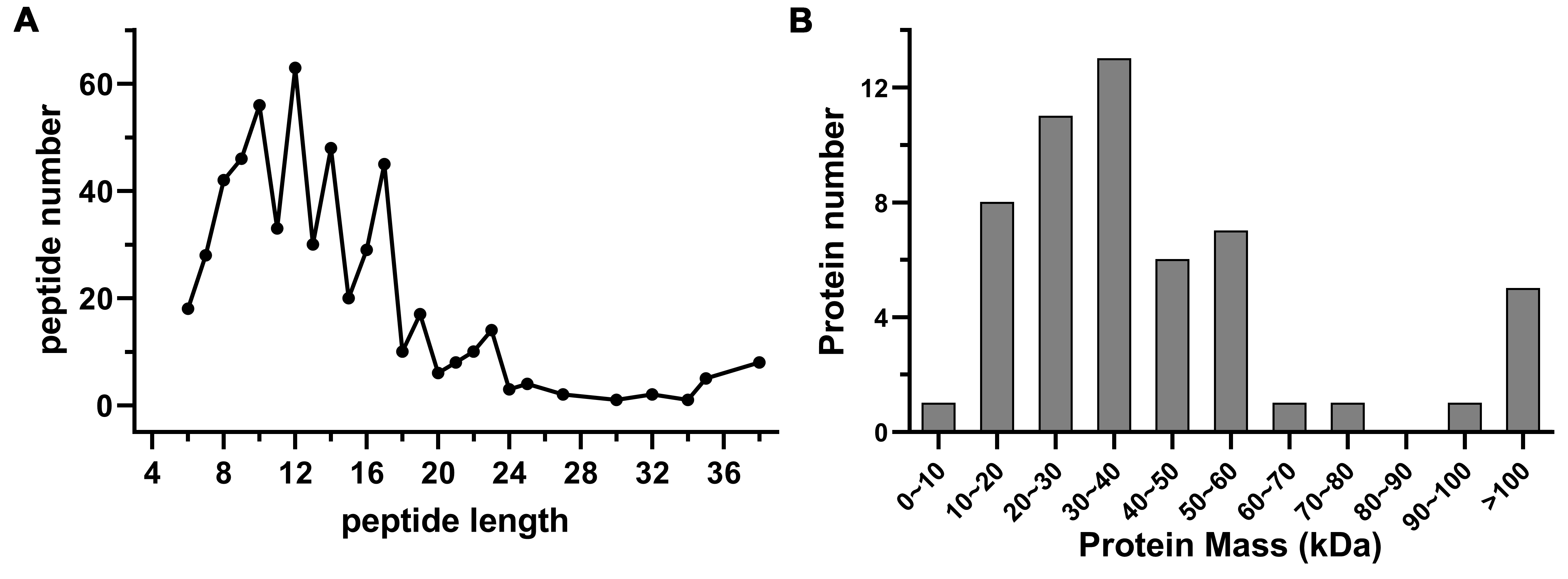

Supplement: Supplementary file 5 [file Image_2.tif]

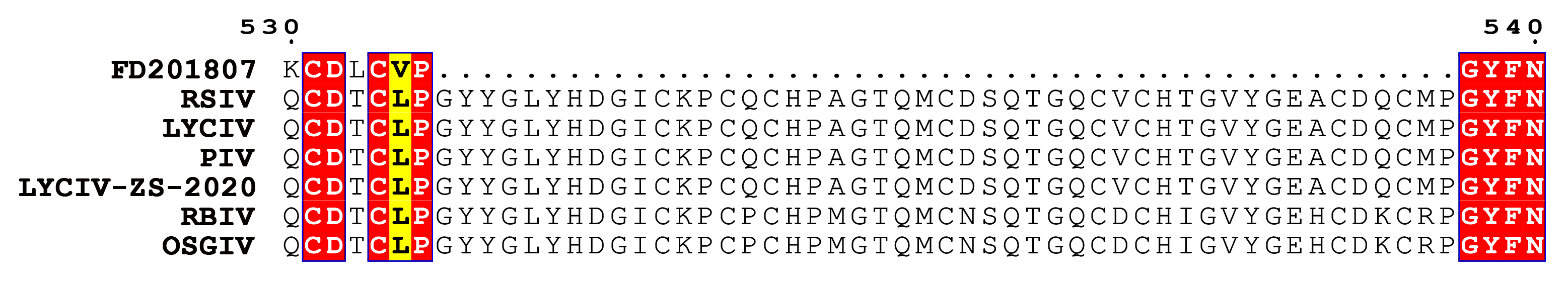

Supplement: Supplementary file 6 [file Image_3.tif]
